# Enhanced conductance response in radio frequency scanning tunnelling microscopy

**DOI:** 10.1038/s41598-022-09820-7

**Published:** 2022-04-13

**Authors:** Bareld Wit, Radovan Vranik, Stefan Müllegger

**Affiliations:** Institute of Semiconductor and Solid State Physics, Johannes Kepler University Linz, 4040 Linz, Austria

**Keywords:** Techniques and instrumentation, Surfaces, interfaces and thin films, Scanning probe microscopy, Surface assembly, Surface spectroscopy

## Abstract

Diverse spectroscopic methods operating at radio frequency depend on a reliable calibration to compensate for the frequency dependent damping of the transmission lines. Calibration may be impeded by the existence of a sensitive interdependence of two or more experimental parameters. Here, we show by combined scanning tunnelling microscopy measurements and numerical simulations how a frequency-dependent conductance response is affected by different DC conductance behaviours of the tunnel junction. Distinct and well-defined DC-conductance behaviour is provided by our experimental model systems, which include C_60_ molecules on Au(111), exhibiting electronic configurations distinct from the well-known dim and bright C_60_’s reported so far. We investigate specific combinations of experimental parameters. Variations of the modulation amplitude as small as only a few percent may result in systematic conductance deviations as large as one order of magnitude. We provide practical guidelines for calibrating respective measurements, which are relevant to RF spectroscopic measurements.

## Introduction

Many spectroscopy methods use coaxial cables as high frequency transmission lines. Their transmission characteristic is strongly frequency dependent, as captured by the transfer function, $T(f)$^1^. Recently, the interest in $T(f)$ has been revived by the development of scanning tunnelling microscopy (STM) towards spin-based spectroscopy of single atoms and molecules, employing radio frequency (RF) modulation of the voltage across the tunnel junction. Different approaches have been reported to date, including the conductance-based detection of single spin (magnetic) resonance^2^, the magnetoresistive detection of electron spin resonance^3–6^ and ferromagnetic resonance^7^, as well as tunnel-current-noise based spin spectroscopy^8^. Most RF-STM methods reported to date measure the tunnelling response while sweeping the modulation frequency, ${f}_{\text{RF}}$. In contrast, some implementations of electron spin resonance STM sweep the magnetic field strength at a fixed microwave frequency^6,9–11^. Both approaches require adequate calibration of the RF voltage amplitude at the tunnel junction, ${V}_{\text{pk,jun}}$^12,13^.

In this work, we show by combined RF-STM experiments and simulations how the result of calibration is strongly affected by the interplay of experimental parameters, including ${V}_{\text{pk,jun}}$, the DC voltage across the tunnel junction, ${V}_{\text{DC}}$, and the local tunnel conductance, $G\left({V}_{\text{DC}}\right)=\partial I({V}_{\mathrm{DC}})/\partial {V}_{\mathrm{DC}}$, where $I({V}_{\mathrm{DC}})$ is the tunnel current. In particular, we show the experimental dependence of $G({f}_{\text{RF}})$ as well as the slope of the conductance—RF amplitude curve, $S\left({V}_{\text{pk,jun}}\right)=\partial G({V}_{\text{pk,jun}})/\partial {V}_{\text{pk,jun}}$, on the above parameters. We find excellent agreement between the results of our experiments and simulations. In all cases investigated herein, $S\left({V}_{\text{pk,jun}}\right)$ is the key to explain the $G({f}_{\text{RF}})$ behaviour. We investigate three model systems, denoted α, β and γ, exhibiting distinct $G\left({V}_{\text{DC}}\right)$. Our results provide guidelines for interpreting RF spectroscopic measurements in general, as well as for $G({f}_{\text{RF}})$ measurements in particular.

## Results

### Model systems with distinct ${\varvec{G}}({{\varvec{V}}}_{\text{DC}})$

**Figure 1:**
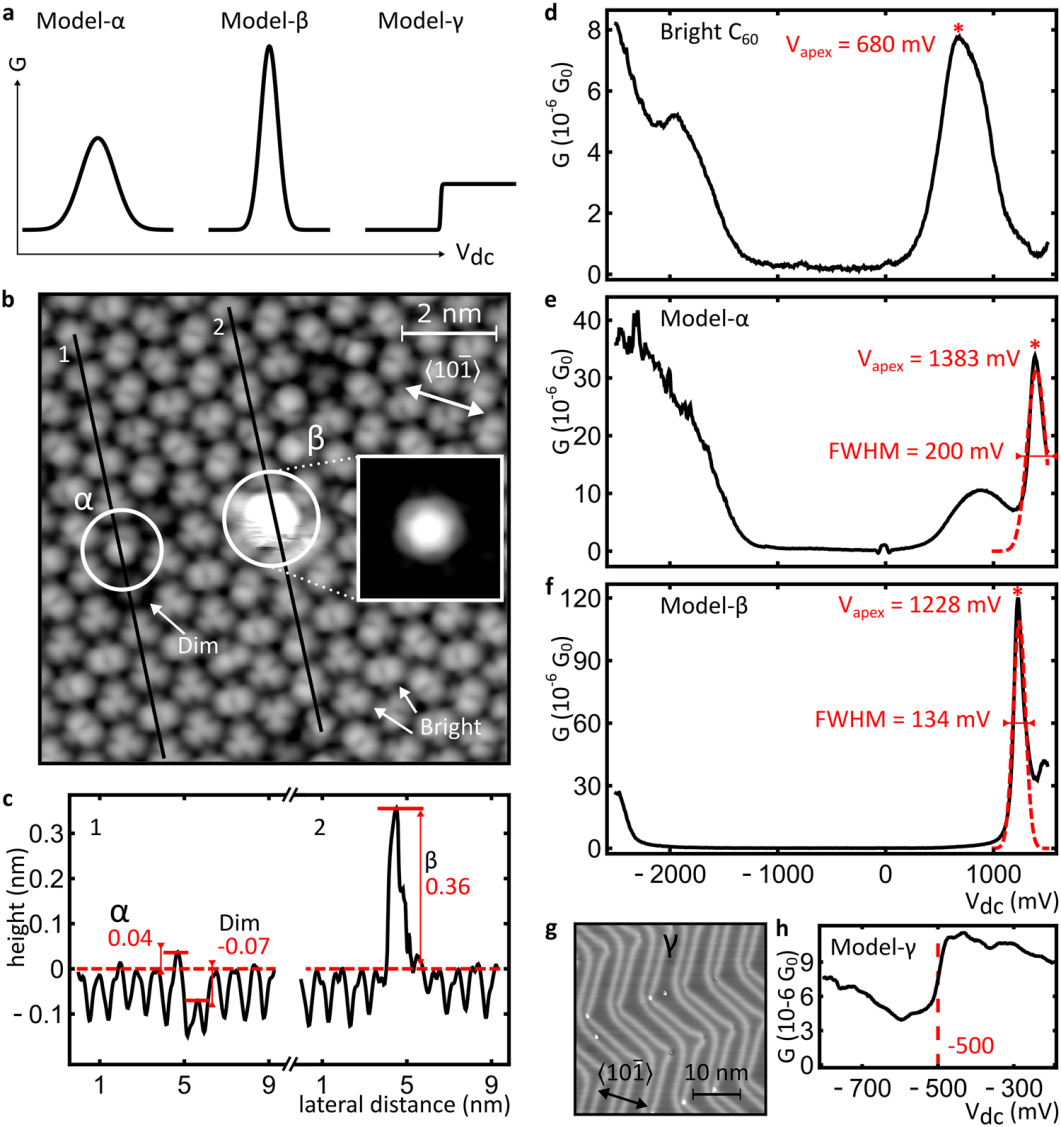
Details of the model systems (**a**) A schematic representation of the different voltage dependences of the conductance of models α, β and γ. (**b**) STM image (0.15 nA; + 1310 mV; 0.25 nm z-scale) of a C_60_ island on Au(111). Example dim and bright C_60_ molecules are indicated with labelled arrows; a model-α and a model-β molecule are indicated with labelled circles. Inset shows an STM image (0.31 nA; + 1273 mV; 0.40 nm z-scale) with contrast optimised to resolve intramolecular details of model-β. (**c**) Height profiles along the black lines as labelled in b. The height values of model-α, model-β and dim molecules relative to bright C_60_ are indicated and dashed lines indicate the average height of bright C_60_. (**d**) Typical $G({V}_{\text{DC}})$ spectrum of bright C_60_. (**e**, **f**) Typical $G({V}_{\text{DC}})$ spectra of model-α and model-β. The red dashed lines indicate Gaussian fits to the sharp peaks used in RF measurements. (**g**) STM image (0.30 nA; + 533 mV; 0.08 nm z-scale) of herringbone-reconstructed Au(111); an fcc region is marked by γ. h) Typical $G({V}_{\text{DC}})$ spectrum of model-γ (average of five spectra; recorded over an fcc region). ${G}_{0}=2e/h\approx 7.75 \cdot {10}^{-5} S$ is the conductance quantum.

We have prepared and characterised three different experimental model systems in a highly reproducible manner, which exhibit distinct $G\left({V}_{\text{DC}}\right)$ behaviour in STM. Specifically, their $G\left({V}_{\text{DC}}\right)$ contains a middle-broad Gaussian-like peak, a sharp Gaussian-like peak and a step, shown schematically in Fig. 1a. Model-α and model-β are specific C_60_ molecules found within sub-monolayer coverage islands of C_60_/Au(111)^14,15^. Model-γ is the clean Au(111) surface^16,17^.

Figure 1b shows a representative STM image of a monolayer island of C_60_/Au(111) at sub-molecular spatial resolution. Within C_60_ islands, the individual molecules are well-known to occur in different configurations. The most common ones are denoted in the literature as bright C_60_ and dim C_60_ respectively, since they appear bright or dim in the STM images^18^. Examples of dim C_60_ and bright C_60_ are labelled as such. As shown in Fig. 1c, dim C_60_ appears about 0.1 nm lower than bright C_60_ in STM, since it adsorbs on an Au surface vacancy site rather than on unreconstructed Au(111)^18^. The different adsorption sites affect the electronic configurations, leading to subtle differences in the empty state $G({V}_{\text{DC}})$ spectra, as reported in the literature^19,20^. Figure 1d shows a representative $G({V}_{\text{DC}})$ of bright C_60_.

We observe two configurations, labelled model-α and model-β in Fig. 1b, that have significantly different $G({V}_{\text{DC}})$ curves, as shown in Figs. 1e,f. In STM, model-α appears as an asymmetric two-lobed shape, where the brighter lobe is about 0.04 nm higher than the common bright C_60_, as shown in Fig. 1c. Its $G({V}_{\text{DC}})$, shown in Fig. 1e, exhibits a peak near ${V}_{\text{apex}}=$ 1375 ± 20 mV as indicated by the red line. Model-β appears as a protrusion which is up to 0.4 nm higher than bright C_60_, as shown in Fig. 1c. Figure 1f shows the $G({V}_{\text{DC}})$ spectrum of model-β, exhibiting a distinct peak near ${V}_{\text{apex}}=$ 1175 ± 75 mV. The large range in ${V}_{\text{apex}}$ is due to distinct β molecules exhibiting a peak at different ${V}_{\text{DC}}$ values within this range. By comparison with literature, the peaks at positive bias in all three C_60_ $G({V}_{\text{DC}})$ spectra can be attributed to C_60_ orbitals derived from the lowest occupied molecular orbitals (LUMO), which are triply degenerate in the gas phase^20,21^. Notice that the full-width at half-maximum (FWHM) of model-β is almost twice as small as that of model-α, while its intensity is about 3 times as high. Moreover, both peaks are significantly narrower and more intense than those observed for ordinary bright C_60_. Since both aspects may significantly impact the RF-STM signal, we focus on the minority species α and β to make use of these exceptionally narrow and intense peaks in the $G({V}_{\text{DC}})$ spectra in our RF measurements.

Model-γ is provided by the characteristic step-like $G({V}_{\text{DC}})$ observed near the onset of the electronic surface-state of the pristine Au(111) surface ^16,17^, as shown in Figs. 1g,h. The step-like $G({V}_{\text{DC}})$ of model-γ is used as a model system, since we routinely use this step and similar step features in $G({V}_{\text{DC}})$ spectra of other metal surfaces to calibrate the RF amplitude at the tunnel junction.

### RF-STM measurements

#### Calibration

We have performed RF-STM measurements on our three different $G({V}_{\text{DC}})$ model systems α, β and γ. First, we use model-γ to calibrate the transmission line to compensate for frequency dependent RF transmission losses. In the vicinity of a non-linearity in a $G({V}_{\text{DC}})$, variations in RF transmission result in systematic differences in conductance^12,22^. Thus, transmission losses are compensated by adjusting the output power of the generator, ${P}_{\text{gen}}$, in such a way that a flat $G\left({f}_{\mathrm{RF}}\right)$ is obtained. A $G\left({f}_{\mathrm{RF}}\right)$ spectrum obtained with a calibrated amplitude is featureless irrespective of $G({V}_{\text{DC}})$ and ${V}_{\mathrm{DC}}$ unless an atom or molecule under the tip shows a microwave resonance. Hence, calibration allows one to distinguish between transmission artefacts and microwave resonance peaks.

#### Effect of $G({V}_{\text{DC}})$ on $G\left({f}_{\text{RF}}\right)$

**Figure 2:**
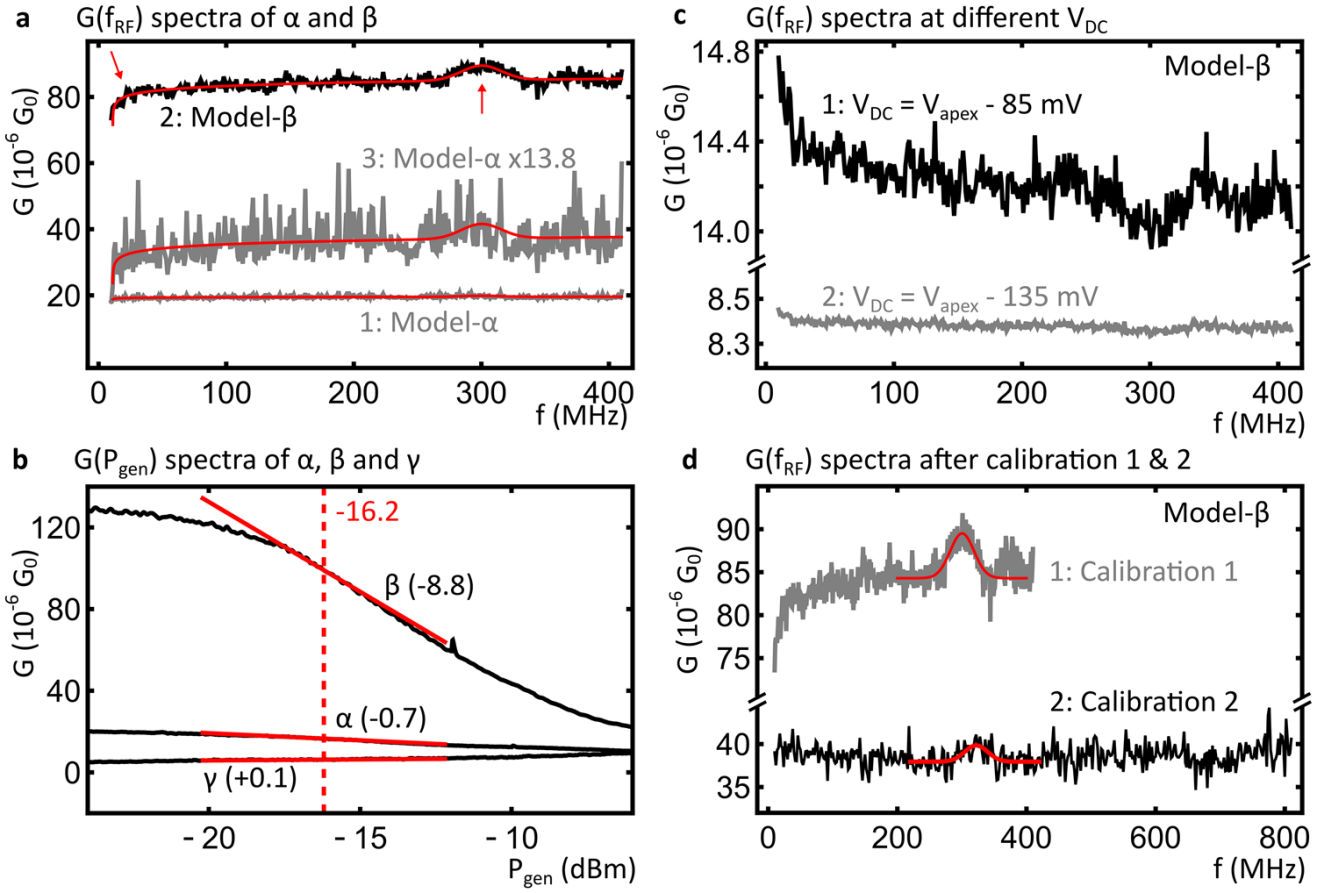
Experimental RF conduction spectra (**a**) A comparison of experimental $G\left({f}_{\text{RF}}\right)$ spectra of models α and β taken at ${V}_{\text{DC}}= {V}_{\text{apex}}$ of their respective $G({V}_{\text{DC}})$ spectra. The scaled (factor 13.8) and vertically offset copy of model-α allows for comparison of spectral details and the red curves indicate numerical fits with Eq. 1 (see text). Red arrows indicate the steady increase between 10 and 80 MHz and a peak at approximately 300 MHz in the spectrum of model-β. (**b**) Experimental $G({P}_{\text{gen}})$ curves of models α, β and γ (average of ten, five and five spectra, respectively), all acquired at constant ${f}_{\text{RF}}=$ 300 MHz. The solid red lines provide a guide to the eye indicating the slope of the curves in units of ${10}^{-6} {\mathrm{G}}_{0}/\mathrm{dBm}$ at ${P}_{\text{gen}}=-16.2$ dBm. (**c**) Experimental $G\left({f}_{\text{RF}}\right)$ spectra of model-β taken at ${V}_{\text{pk,jun}}=$ 25 mV and ${V}_{\text{DC}}= {V}_{\text{apex}}-$ 85 mV (1) or ${V}_{\text{DC}}= {V}_{\text{apex}}-$ 135 mV (2), below the inflection point of the $G({V}_{\text{DC}})$ peak. d) A comparison of experimental $G\left({f}_{\text{RF}}\right)$ spectra of model-β with calibrated ${V}_{\text{pk,jun}}$ taken at ${V}_{\text{DC}}= {V}_{\text{apex}}$ of their respective $G({V}_{\text{DC}})$ spectra. Red curves indicate numerical fits with Gaussian functions to the peaks at approximately 300 and 326 MHz, respectively. ${\mathrm{G}}_{0}=2e/h\approx 7.75 \cdot {10}^{-5} S$ is the conductance quantum.

On models α and β we measure $G\left({f}_{\text{RF}}\right)$ spectra with a calibrated, frequency independent, ${V}_{\text{pk,jun}}=$ 100 mV. Figure 2a illustrates the effect of different shapes of $G({V}_{\text{DC}})$ on $G\left({f}_{\text{RF}}\right)$ spectra. To allow for comparison, all spectra shown in Fig. 2a were obtained at equivalent values of ${V}_{\text{DC}}$, namely at the ${V}_{\text{apex}}$ (see Figs. 1e,f). Curve 1 (model-α) in Fig. 2a, looks almost featureless; without further analysis one may conclude that model-α does not show a frequency dependent response and that ${V}_{\text{pk,jun}}$ is properly calibrated. In contrast, curve 2 (model-β) exhibits a peak around ${f}_{\text{RF}}=$ 300 MHz as well as a steady increase between 10 and 80 MHz, as indicated by the arrows in Fig. 2a. Without further analysis, one might misinterpret this peak as a resonance response to the RF modulation, similar to a peak in the rectification current^13^.

#### *The role of*$G({P}_{\mathrm{gen}})$

Detailed analysis shows that the different shapes of curves 1 and 2 in Fig. 2a can be attributed to the underlying model system. In particular, it is explained by how the combination of $G({V}_{\mathrm{DC}})$ spectra and ${V}_{\mathrm{DC}}$ affect the relation between the measured conductance and the RF amplitude. This relation is shown in Fig. 2b as $G({P}_{\mathrm{gen}})$ curves of α and β taken at the same $G({V}_{\mathrm{DC}})$ as the $G\left({f}_{\mathrm{RF}}\right)$ spectra. These $G({P}_{\mathrm{gen}})$ curves relate the measured conductance directly to the output power of the generator, which is the experimentally adjustable parameter controlling the RF amplitude at the tunnel junction. The slopes of the three $G({P}_{\mathrm{gen}})$ spectra at the setpoint bias are clearly different for the three model systems. Thus, a change in RF amplitude will not result in the same change in $G$ when measured over different models.

Specifically, any small deviation in ${V}_{\text{pk,jun}}({f}_{\text{RF}})$ translates to deviations in $G\left({f}_{\text{RF}}\right)$ according to the slope of $G$ with respect to ${V}_{\text{pk,jun}}$:

1 $$G\left( {f_{{RF}} } \right) - \left\langle {G\left( {f_{{RF}} } \right)} \right\rangle = S\left( {\left\langle {V_{{pk,jun}} \left( {f_{{RF}} } \right)} \right\rangle } \right) \cdot \left( {V_{{pk,jun}} \left( {f_{{RF}} } \right) - \left\langle {V_{{pk,jun}} \left( {f_{{RF}} } \right)} \right\rangle } \right)$$

Here, $\langle {V}_{\text{pk,jun}}\left({f}_{\text{RF}}\right)\rangle$ is the mean value ${V}_{\text{pk,jun}}\left({f}_{\text{RF}}\right)$, which is 100 mV in our experiments. Likewise, $\langle G\left({f}_{\text{RF}}\right)\rangle$ is the average value of $G\left({f}_{\text{RF}}\right)$, which is determined by $G({V}_{\text{DC}})$, ${V}_{\text{DC}}$ and $\langle {V}_{\text{pk,jun}}\left({f}_{\text{RF}}\right)\rangle$. $S\left({V}_{\text{pk,jun}}\right)=\partial G\left({V}_{\text{pk,jun}}\right)/\partial {V}_{\text{pk,jun}}$ is the slope of the corresponding $G\left({V}_{\text{pk,jun}}\right)$ spectrum, and should be evaluated at ${V}_{\text{pk,jun}}=\langle {V}_{{\text{pk,jun}}}\left({f}_{\text{RF}}\right)\rangle$. The slope for model-α is about 12.6 times smaller than for model-β. Thus, model-β is considerably more sensitive to any small variation in RF amplitude which may still persist after calibration.

We use Eq. 1, in combination with an analytic function, ${V}_{\text{pk,jun}}^{sim}\left({f}_{\text{RF}}\right)$, that resembles the shape of curve 2 of Fig. 2a, to fit the experimental $G\left({f}_{\text{RF}}\right)$ spectra. The amplitude of the deviations in ${V}_{\text{pk,jun}}({f}_{\text{RF}})$ was taken to be 2.5 mV, in accordance with careful measurements of ${V}_{\text{pk,jun}}$ performed on model-β^12^. The only fit parameter was the slope, $S(\langle {V}_{\text{pk,jun}}\left({f}_{\text{RF}}\right)\rangle )$. The resulting fits are depicted as red curves in Fig. 2a. From this procedure, we obtain a ratio between the slopes on models α and β of 13.8, which is very close to the experimentally obtained ratio of 12.5. To further illustrate the agreement, similar features are present in the $G\left({f}_{\text{RF}}\right)$ spectrum of model-α, multiplied by 13.8, shown in Fig. 2a curve 3, and the spectrum taken over model-β.

In order to directly compare the slopes obtained from the fit, 0.13∙10^–6^ G_0_/mV and 1.77∙10^–6^ G_0_/mV for models α and β respectively, and the slopes of the $G({P}_{\text{gen}})$ curves of Fig. 2b, the latter have to be converted to $G\left({V}_{\text{pk,jun}}\right)$. This yields slopes of approximately 0.06∙10^–6^ G_0_/mV and 0.7∙10^–6^ G_0_/mV, in good agreement with the fits. The remaining difference can be attributed to uncertainties in the magnitude of the small deviations in ${V}_{\text{pk,jun}}({f}_{\text{RF}})$ as well as the value of the $T({f}_{\text{RF}})$ required for the conversion between ${P}_{\text{gen}}$ and ${V}_{\text{pk,jun}}$.

Additionally, Fig. 2b shows the $G({P}_{\text{gen}})$ spectrum of model-γ. In this case, the slope is even smaller. Thus, the sensitivity of $G\left({f}_{\text{RF}}\right)$ towards small changes in ${V}_{\text{pk,jun}}$ during calibration is worse than during measurements on α and β. This explains why small variations in ${V}_{\text{pk,jun}}$ persist after calibration: they are too small to detect on model-γ. In general, it is important to note that the slope of $G\left({V}_{\text{pk,jun}}\right)$, $S\left({V}_{\text{pk,jun}}\right)$, is not a property of the measurement set-up, but rather a property of the sample; it depends on the electronic structure of the sample (and tip), $G({V}_{\text{DC}})$, in conjunction with the setpoint bias, ${V}_{\text{DC}}$, at which it is measured.

#### Effect of ${V}_{\text{DC}}$ on $G\left({f}_{\text{RF}}\right)$

Another consequence of the dependence of $S\left({V}_{\text{pk,jun}}\right)$ on measurement parameters is highlighted in Fig. 2c, which shows two additional $G\left({f}_{\text{RF}}\right)$ spectra taken on model-β, only taken at ${V}_{\text{DC}}$ below the inflection point of the peak in $G({V}_{\text{DC}})$. Note that these spectra were obtained with a ${V}_{\text{pk,jun}}=$ 25 mV in order to avoid including the entire peak in $G({V}_{\text{DC}})$; we carefully checked that at ${V}_{\text{DC}}={V}_{\text{apex}}$ the shape of the $G\left({f}_{\text{RF}}\right)$ spectra did not change when we reduced ${V}_{\text{pk,jun}}$ from 100 to 25 mV. Both spectra resemble curve 1 in Fig. 2a, except that they are inverted: the initial increase is now a decrease and where there was a peak, there is now a dip in conductance. Indeed, at the apex of a $G({V}_{\text{DC}})$ peak, the apparent $G$ decreases with increasing RF amplitude, as shown in Fig. 2b, whereas near the base of a $G({V}_{\text{DC}})$ peak, the apparent $G$ increases. Thus, $S\left({V}_{\text{pk,jun}}\right)$ changes sign and the $G\left({f}_{\text{RF}}\right)$ spectrum inverts. Note that the magnitude of the slope also changes with ${V}_{\text{DC}}$, which explains the stark difference in the two spectra shown in Fig. 2c.

#### Approximations to $G({P}_{\text{gen}})$ in calibration

Figure 2d compares $G\left({f}_{\text{RF}}\right)$ spectra taken on model-β with the original calibration and a second, independent, calibration. In the second calibration, a different approximation was used in the calculation of the required adjustment in ${P}_{\text{gen}}$. After the second calibration, the initial sharp increase in the $G\left({f}_{\text{RF}}\right)$ spectrum is no longer present and only a small peak near ${f}_{\text{RF}}=$ 326 MHz is observed, as indicated with the red curves. Crucially, the differences in the two calibration results correlated directly with the changes in the $G\left({f}_{\text{RF}}\right)$ spectrum. This shows that the measurement outcome can be calibration dependent, even if both calibration procedures resulted in sufficiently flat $G\left({f}_{\text{RF}}\right)$ traces measured on model-γ. Here, the new approximation resulted in a better performance, *i. e.* smaller variations in ${V}_{\text{pk,jun}}$.

### Simulation of $S\left({V_{{{\text{pk,jun}}}}} \right)$

Numerical simulations were performed, motivated by the insight gained from the experimental results. Specifically, idealised ${G}^{sim}({V}_{\text{DC}})$ spectra only containing a single sigmoid function step or a single Gaussian peak, as shown in Fig. 1a, were used to obtain ${S}^{sim}\left({V}_{\text{pk,jun}}\right)$, as described in the methods. These simulations enable the systematic investigation of how ${S}^{sim}\left({V}_{\text{pk,jun}}\right)$ is influenced by parameters, such as the height and width of the $G({V}_{\text{DC}})$ step/peak, ${V}_{\text{pk,jun}}$, and ${V}_{\text{DC}}$.

A key result derived from our simulations is that ${S}^{sim}\left({V}_{\text{pk,jun}}\right)$ scales linearly with the height of the peak or step in the ${G}^{sim}({V}_{\text{DC}})$ spectrum. This explains why the sensitivity towards ${V}_{\text{pk,jun}}$ is highest for model-β and lowest for model-γ: the intensity of the peak in model-β is about three times higher than the peak of model-α and over ten times higher than the step in model-γ. Thus, the experimentally observed intensity of a $G({V}_{\text{DC}})$ peak/step is a strong quantitative measure of how sensitive the $G\left({f}_{\text{RF}}\right)$ spectrum depends on ${V}_{\text{pk,jun}}$.

**Figure 3:**
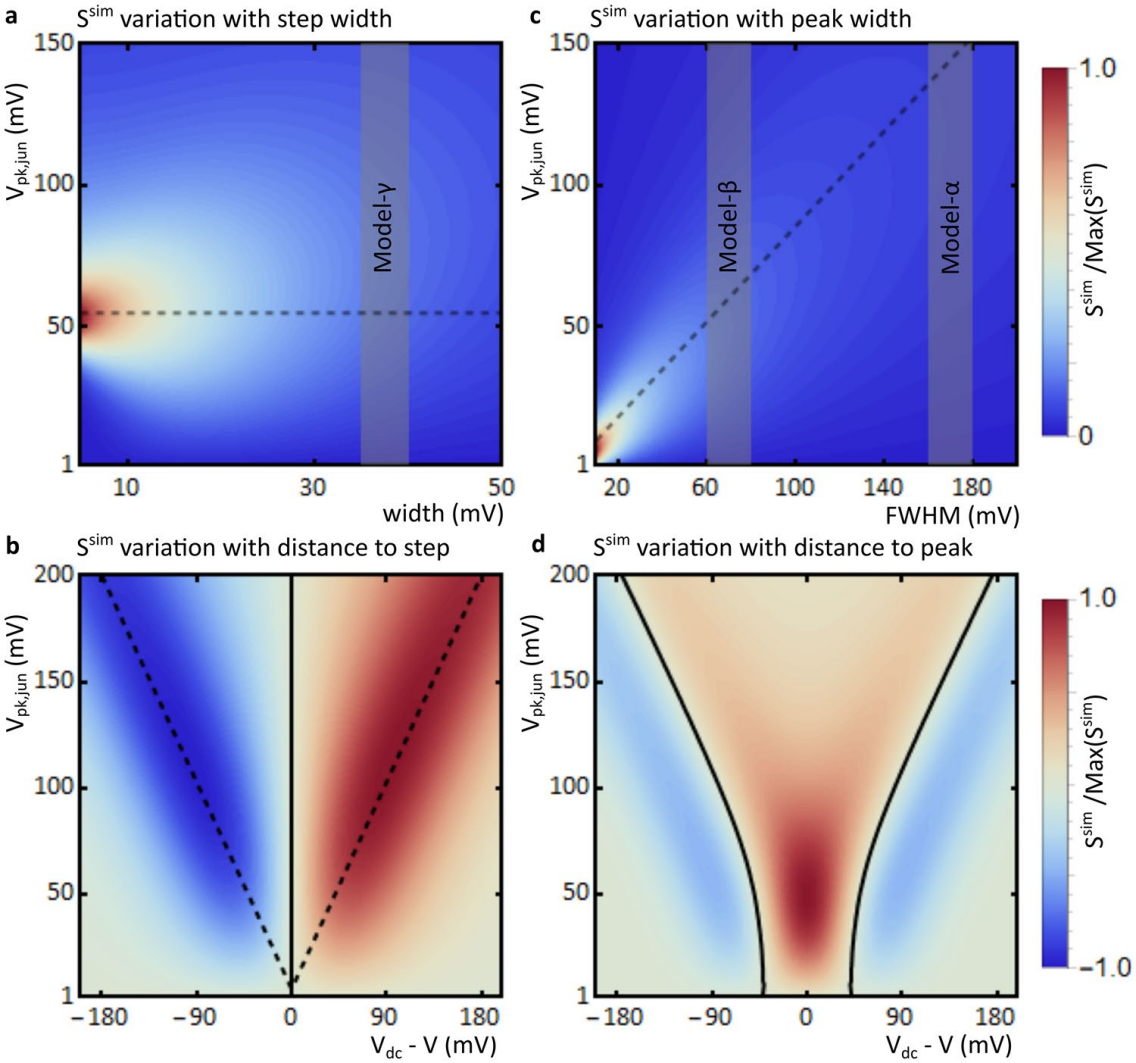
Simulated parameter maps (**a**) A parameter map of normalised $\left|{S}^{sim}\left({V}_{\text{pk,jun}},\mathrm{ width}\right)\right|$ for a sigmoid function step ${G}^{sim}({V}_{\text{DC}})$ spectrum at ${V}_{\text{DC}}=$ -50 mV. Red colours correspond to maximum sensitivity towards ${V}_{\text{pk,jun}}$. The grey area indicates the width of the experimental model-γ and the dashed line corresponds to a value of ${V}_{\text{pk,jun}}=\left|{V}_{\text{DC}}\right|+$ 5 mV. (**b**) A parameter map of normalised ${S}^{sim}\left({V}_{\text{pk,jun}}, {V}_{\mathrm{DC}}\right)$ for a sigmoid function step ${G}^{sim}({V}_{\text{DC}})$ spectrum at a step width of 37 mV. Red and blue colours correspond to maximum positive and negative sensitivity towards ${V}_{\text{pk,jun}}$, respectively. The solid black line represents zero sensitivity and the dashed line again corresponds to a value of ${V}_{\text{pk,jun}}=\left|{V}_{\text{DC}}\right|+$ 5 mV. (**c**) A parameter map of normalised $\left|{S}^{sim}\left({V}_{\text{pk,jun}},\mathrm{FWHM}\right)\right|$ for a Gaussian peak ${G}^{sim}({V}_{\text{DC}})$ spectrum at ${V}_{\text{DC}}=$ 0 mV. The grey areas indicate the FWHM of the experimental models β and α and the dashed line corresponds to a value of ${V}_{\text{pk,jun}}=$ 0.85∙FWHM. (**d**) A parameter map of ${S}^{sim}\left({V}_{\text{pk,jun}}, {V}_{\mathrm{DC}}\right)$ for a Gaussian peak ${G}^{sim}({V}_{\text{DC}})$ spectrum at a FWHM of 70 mV. The solid black line represents zero sensitivity.

The width of the step or peak may also have a significant effect. Figure 3a,c show parameter maps of normalised $\left|{S}^{sim}\left({V}_{\text{pk,jun}},\mathrm{width}\right)\right|$ for a step and a peak, highlighting the differences between the two. As indicated by the dashed line in Fig. 3a, in the case of a sharp step, the maximum $\left|{S}^{sim}\left({V}_{\text{pk,jun}}\right)\right|$ is obtained for ${V}_{\text{pk,jun}}\approx \left|{V}_{\text{DC}}\right|+$ 5 mV and with increasing width, the maximum ${S}^{sim}\left({V}_{\text{pk,jun}}\right)$ only gradually shifts to higher values of ${V}_{\text{pk,jun}}$. Conversely, as indicated by the dashed line in Fig. 3c, in the case of a peak the maximum of ${S}^{sim}\left({V}_{\text{pk,jun}}\right)$ is obtained for ${V}_{\text{pk,jun}}\approx$ 0.85∙FWHM. In either case, a higher maximum $\left|{S}^{sim}\left({V}_{\text{pk,jun}}\right)\right|$ is obtained for sharper steps or narrower peaks. In the case of relatively broad steps or peaks, $\left|{S}^{sim}\left({V}_{\text{pk,jun}}\right)\right|$ is more uniform and the dependence on ${V}_{\text{pk,jun}}$ is less pronounced. The widths of experimental models α, β and γ are indicated in the plots by grey areas. Since the FWHM of the $G({V}_{\text{DC}})$ peak of β is smaller than that of α, ${S}^{sim}\left({V}_{\text{pk,jun}}\right)$ depends more strongly on ${V}_{\text{pk,jun}}$ in the case of β. The simulations also shows that $\left|{S}^{sim}\left({V}_{\text{pk,jun}}\right)\right|$ is generally smaller for steps than for peaks of the same height, as the values are higher prior to normalisation for the latter than for the former, further explaining why the sensitivity towards ${V}_{\text{pk,jun}}$ is higher for models α and β than for γ.

Figure 3b shows a parameter map of ${S}^{sim}\left({V}_{\text{pk,jun}},{V}_{\text{DC}}\right)$ for steps at a width corresponding to experimental model-γ. Since ${V}_{\text{pk,jun}}$ and ${V}_{\text{DC}}$ are chosen in experiments, this map can be used to optimise calibrations. In the case of a step function, ${S}^{sim}\left({V}_{\text{pk,jun}}\right)$ is always zero at the inflection point of the step (here at ${V}_{\text{DC}}=$ 0 mV). As indicated by the dashed line in Fig. 3b, $\left|{S}^{sim}\left({V}_{\text{pk,jun}}\right)\right|$ is highest for a value of ${V}_{\text{pk,jun}}\approx \left|{V}_{\text{DC}}\right|+$ 5 mV. Thus, in order to improve calibration using conductance measurements, it is important to choose a value of ${V}_{\text{DC}}$ close to the step, but not too close; for experimental model-γ between 50 and 100 mV away from the step is appropriate. The target ${V}_{\text{pk,jun}}$ should be slightly larger than the distance between ${V}_{\text{DC}}$ and the step, as to include a significant number of points across the step.

Figure 3d shows a parameter map of ${S}^{sim}\left({V}_{\text{pk,jun}},{V}_{\text{DC}}\right)$ for peaks at a FWHM corresponding to experimental model-β. For a peak, the maximum $\left|{S}^{sim}\left({V}_{\text{pk,jun}}\right)\right|$ is obtained when ${V}_{\text{DC}}$ coincides with the top of the peak and ${V}_{\text{pk,jun}}$ is slightly less than the FWHM of the peak. Additionally, when moving away from the peak, ${S}^{sim}\left({V}_{\text{pk,jun}}\right)$ decreases until it becomes zero, as indicated by the black line in Fig. 3d. This way, ${S}^{sim}\left({V}_{\text{pk,jun}}\right)$ can be minimised for $G\left({f}_{\text{RF}}\right)$ spectra. When $\left|{V}_{\text{DC}}\right|$ is increased further, ${S}^{sim}\left({V}_{\text{pk,jun}}\right)$ becomes negative. This is consistent with the inversion observed experimentally for $G\left({f}_{\text{RF}}\right)$ spectra near the base of the peak of model-β described above.

While generally the properties of $G({V}_{\text{DC}})$ cannot be freely varied in most experimental situations, our simulation results show that by careful choice of the values of ${V}_{\text{DC}}$ and ${V}_{\text{pk,jun}}$, the sensitivity towards ${V}_{\text{pk,jun}}$ can be optimised. This can be exploited to improve the calibration procedure, by first finding the optimal combination of ${V}_{\text{pk,jun}}$ and ${V}_{\text{DC}}$ for a given $G({V}_{\text{DC}})$ spectrum, or adjusting them to reduce sensitivity in $G\left({f}_{\text{RF}}\right)$ measurements.

## Conclusion

In this work, we have used STM experiments and numerical simulations to investigate how certain differences in the DC electrical conductance $G({V}_{\text{DC}})$ affect the frequency-dependent conductance response under periodic modulation of the bias voltage at MHz to GHz frequencies. Our experimental model systems include C_60_/Au(111) exhibiting electronic configurations distinct from the well-known dim and bright C_60_’s reported so far. We show that, at specific parameter combinations, variations of the modulation amplitude as small as only a few percent may result in systematic conductance deviations as large as one order of magnitude. We provide practical guidelines for calibrating respective measurements, which are relevant to RF spectroscopy, in general, as well as for $G({f}_{\text{RF}})$ measurements, in particular.

## Methods

Sample preparation was performed in an ultra-high vacuum (UHV) system with base pressure < 4∙10^–10^ mbar. The Au(111) crystal (SPL) was prepared by repeated cycles of Ar ion sputtering (0.61 kV, 10 min) and annealing (703 K, 10 min). The cleanliness of the pristine Au(111) surface was confirmed by STM imaging prior to the deposition of molecules. C_60_ powder (Acros Organics; 99.9% pure) was degassed prior to deposition (723 K, 20 min). A C_60_ layer with a nominal coverage of about 0.1 monolayers was prepared by thermal sublimation from a home-built quartz evaporator with the Au(111) crystal at room temperature and at a pressure of < 2∙10^–9^ mbar. The temperature of the evaporator was permanently monitored with a K-type thermocouple inside the quartz evaporator. The deposition has been carried out in consecutive steps as follows: deposition with source temperature of 773 K for 7 h, deposition with source temperature of 763 K for 24 h, annealing at 423 K for 10 min, deposition with source temperature of 763 K for 24 h, and annealing at 323 K for 10 min. During deposition, a mask was used such that only part of the Au(111) surface was exposed to the molecular beam, in order to keep areas of pristine Au(111). The sample was transferred into the STM chamber without breaking the vacuum.

**Figure 4:**
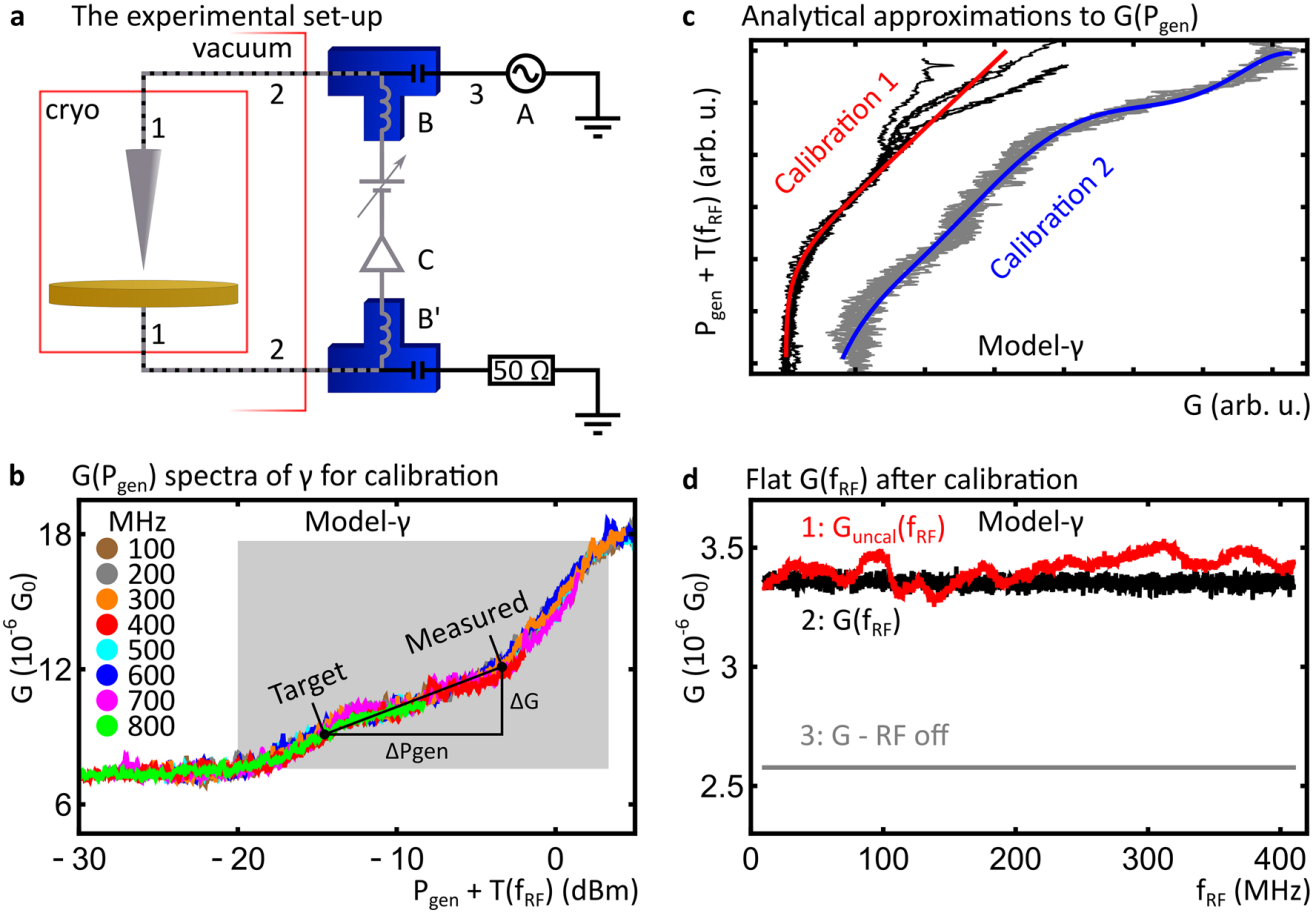
Details of the set-up and calibration (**a**) A schematic of the RF-STM set-up. (**b**) $G({P}_{\text{gen}})$ spectra obtained on model-γ (${V}_{\text{DC}}=$ -550 mV) for eight different ${f}_{RF}$ values. Spectra are shifted horizontally to account for differences in $T({f}_{RF})$. (**c**) Inverted $G({P}_{\text{gen}})$ spectra obtained on model-γ (${V}_{\text{DC}}=$ -530 mV and ${V}_{\text{DC}}=$ -550 mV for the black and grey spectra, respectively) and their respective analytical approximation, used for calibration 1 and 2. (**d**) $G\left({f}_{\text{RF}}\right)$ spectra with RF modulation on, uncalibrated (1) and calibrated (2), obtained on model-γ with constant ${V}_{\text{DC}}=$ -530 mV. The nominal value of G in absence of RF modulation is also indicated (3).

STM experiments were performed at a base pressure of < 4∙10^–11^ mbar and a temperature of < 8.5 K on our RF-upgraded Createc low temperature STM, shown schematically in Fig. 4a^2,22,23^. To allow for RF modulation of the tunnel voltage, a sinusoidal voltage of frequency ${f}_{\text{RF}}$ from an RF generator (Keysight N5173B, component A in Fig. 4a) is added to ${V}_{\mathrm{DC}}$ via a bias-tee (SHF BT45R-B, component B in Fig. 4a) and fed to the STM tip. The sample is connected via a separate bias-tee (Tektronix PSPL5541A, component B’ in Fig. 4a) where the DC line is grounded via the tunnel current amplifier (Femto DLPCA 200, component C in Fig. 4a) and the RF line is fed into a 50 Ω load. RF transmission occurs via RF rated transmission cables inside the vacuum (Elspec MK5001 and Elspec Stormflex 047Cryo, components 1 and 2 in Fig. 4a, respectively) and between generator and the bias tee (Micable B04-40-48-4 M, component 3 in Fig. 4a).

Experimental measurement of $G$ was done by an external lock in amplifier (EG&G 5210) using sinusoidal modulation of ${V}_{\mathrm{DC}}$ (${V}_{{\text{rms}},\mathrm{lock}-\mathrm{in}}=$ 12 mV, ${f}_{\text{lock-in}}=$ 775 Hz). Notice that, in general, $G=G({V}_{\mathrm{DC}},{P}_{\text{gen}},{f}_{\text{RF}})$, but when we measure $G$ as a function of one of these variables, we denote it $G({V}_{\mathrm{DC}})$, $G\left({P}_{\text{gen}}\right)$ or $G\left({f}_{\text{RF}}\right)$. Experimental $G({V}_{\mathrm{DC}})$ spectra were obtained in constant-height mode (STM feedback off) and with a typical acquisition time of 40 to 240 s. $G\left({P}_{\text{gen}}\right)$ is obtained experimentally at fixed ${V}_{\mathrm{DC}}$ and fixed ${f}_{\text{RF}}$ while sweeping ${P}_{\text{gen}}$ with 1/30 dB steps from -30 to 0 dBm. Each step was integrated for 0.1 s, resulting in a total acquisition time of 90 s. $G\left({P}_{\text{gen}}\right)$ spectra were taken in constant-current mode (STM feedback on), with a tunnelling current setpoint of 0.3 nA, unless otherwise stated. Since ${P}_{\mathrm{gen}}$ is related to the RF amplitude ${V}_{\mathrm{pk},\mathrm{jun}}$ according to:

$$V_{{{\text{pk}},{\text{jun}}}} = \sqrt {10^{{\left( {\frac{{P_{{{\text{gen}}}} + T\left( {f_{{{\text{RF}}}} } \right)}}{10} - 1} \right)}} }$$

$G\left({P}_{\text{gen}}\right)$ curves can be converted to $G\left({V}_{\mathrm{pk},\mathrm{jun}}\right)$ curves if the transfer function at the measurement frequency is known.

Notice that experimental $G({f}_{\text{RF}})$ is measured in two different ways: uncalibrated and calibrated. In this work $G\left({f}_{\text{RF}}\right)$ refers to calibrated spectra, whereas uncalibrated spectra are denoted ${G}_{uncal}\left({f}_{\text{RF}}\right)$. Uncalibrated means to maintain ${P}_{\text{gen}}({f}_{\text{RF}})=$ constant, causing ${V}_{\text{pk,jun}}({f}_{\text{RF}})$ to be frequency dependent. Calibrated means to properly vary ${P}_{\text{gen}}({f}_{\text{RF}})$ in order to obtain frequency-independent (constant) ${V}_{\text{pk,jun}}$^22^. The value of the constant ${V}_{\text{pk,jun}}$ during $G({f}_{\text{RF}})$ measurements is determined independently by measuring $G({V}_{\text{DC}})$ with and without RF modulation, as described in Reference 22. A desired ${V}_{\text{pk,jun}}$ can be achieved by adding/subtracting an appropriate constant from ${P}_{\text{gen}}({f}_{\text{RF}})$. Both ${G}_{uncal}\left({f}_{\text{RF}}\right)$ and calibrated $G\left({f}_{\text{RF}}\right)$ are measured at fixed ${V}_{\text{DC}}$ while sweeping ${f}_{\text{RF}}$ during a total acquisition time of 400 s. $G\left({f}_{\text{RF}}\right)$ spectra were taken in constant-current mode (STM feedback on), with a tunnelling current setpoint of 0.3 nA, unless otherwise stated. On α and β, $G\left({f}_{RF}\right)$ spectra were taken at ${V}_{\text{apex}}$, which was carefully determined from $G({V}_{\mathrm{DC}})$ spectra taken right before the RF measurement, unless otherwise stated. To minimise time dependent artefacts in the experimental $G\left({f}_{RF}\right)$, we randomised the order of the ${f}_{RF}$ values during the measurement of $G\left({f}_{RF}\right)$ on C_60_.

${G}_{uncal}\left({f}_{\text{RF}}\right)$ depends non-linearly on $T({f}_{\mathrm{RF}})$, since for a constant ${P}_{\text{gen}}({f}_{\text{RF}})$ the transfer function determines the RF power at the junction, ${P}_{\text{jun}}({f}_{\text{RF}})$^22^. Unlike $T({f}_{\mathrm{RF}})$, which is only a property of the transmission line characteristics, the details of a ${G}_{uncal}\left({f}_{\text{RF}}\right)$ spectrum depend on the conductance of the tunnel junction $G({V}_{\mathrm{DC}})$ and setpoint bias ${V}_{\mathrm{DC}}$ as well as the transfer function. Thus ${G}_{uncal}\left({f}_{\text{RF}}\right)$ and $T({f}_{\mathrm{RF}})$ are not equivalent quantities. We found no significant change of ${G}_{uncal}\left({f}_{\text{RF}}\right)$ over more than 4 weeks, nor in response to changes in external parameters such as cryostat filling levels, highlighting the stable $T({f}_{\mathrm{RF}})$ of our RF-STM setup. The non-linear dependence of ${G}_{uncal}\left({f}_{\text{RF}}\right)$ on $T({f}_{\mathrm{RF}})$ is equivalently captured by $G\left({P}_{\text{gen}}\right)$, since ${{P}_{\text{jun}}({f}_{\mathrm{RF}}) =P}_{\text{gen}}({f}_{\mathrm{RF}}) +T({f}_{\text{RF}})$. In essence, a $G\left({P}_{\text{gen}}\right)$ spectrum samples the broadening of the surface state step feature as a function of RF amplitude at the chosen constant bias voltage value. Thus, $G\left({P}_{\text{gen}}\right)$, measured with equal $G({V}_{\mathrm{DC}})$ and at the same ${V}_{\mathrm{DC}}$ used for ${G}_{uncal}\left({f}_{\text{RF}}\right)$ spectra, is used as the basis for the calibration procedure. In this work, calibration was performed on model-γ, at a ${V}_{\text{DC}}$ 30 to 50 mV away from the electronic surface state of Au(111) at −500 mV^16,17^, since in this ${V}_{\text{DC}}$ range the change in G with RF amplitude is greatest.

As described in Reference 12, calibration requires that the shape of the $G\left({P}_{\text{gen}}\right)$ curve is independent of ${f}_{\text{RF}}$, while it tolerates constant shifts of the $G\left({P}_{\text{gen}}\right)$ curve with respect to ${P}_{\text{gen}}$ due to $T({f}_{\mathrm{RF}})$. Prior to our RF-STM experiments, we carefully confirmed that $G\left({P}_{\text{gen}}\right)$ spectra, shifted by $T({f}_{\mathrm{RF}})$, obtained on model-γ are indeed independent of ${f}_{\text{RF}}$, see Fig. 4b. Note that the initial rise in G, between -20 and -12 dBm, is due to the RF amplitude crossing the step; the further rise in G at higher amplitudes can be attributed to non-linearities in the $G({V}_{\mathrm{DC}})$ spectrum away from the step. Moreover, with a stable STM tip we found no significant change of the shape of $G\left({P}_{\text{gen}}\right)$ after several days. During calibration the STM tip is required to be sufficiently stable to guarantee consistent shape of $G\left({P}_{\text{gen}}\right)$. After finishing, the obtained calibration, and following RF-STM measurements based on it, are robust against possible tip changes. Additionally, to allow for unambiguous mapping of $\Delta G$ into ${\Delta P}_{\text{gen}}$, $G\left({P}_{\text{gen}}\right)$ is required to be bijective in the RF amplitude range used for calibration. As clearly shown in the shaded area of Fig. 4b, between -20 and 3 dBm, this requirement is satisfied.

Efficient calibration requires the representation of ${P}_{\text{gen}}\left(G\right)$ as an analytical function. Here, we obtain these analytical functions in two different ways and compare their effect on calibration (see main text). For the first calibration, the initial rise, between -23 and -13 dBm, in the experimental $G\left({P}_{\text{gen}}\right)$ is numerically fitted with the function $-A \cdot B \cdot {P}_{gen}/\left({e}^{-B \cdot {P}_{gen}}-1\right)$, where $A$ and $B$ are fit parameters, followed by inversion of the function. For the second calibration, the inverted data $P_{{{\text{gen}}}} \left( G \right)$ is fitted with a 6th order polynomial function. The two functions represent different ways to approximate the experimentally observed $P_{{{\text{gen}}}} \left( G \right)$ curves. Figure 4c shows the comparison of these approximations with their respective $P_{{{\text{gen}}}} \left( G \right)$ spectra. Calibration is achieved by calculating the required adjustment in $P_{{{\text{gen}}}}$, $\Delta P_{{{\text{gen}}}}$, according to the difference between the measured $G$ and the target value of $G$, $\Delta G$, using the respective analytic approximation to $P_{{{\text{gen}}}} \left( G \right)$, as illustrated in Fig. 4b. Typically, two to three iterations of this procedure are performed. Figure 4d compares $G$ measured without RF modulation against ${G}_{uncal}\left({f}_{\text{RF}}\right)$ and $G\left({f}_{RF}\right)$. For reference, a plot of $T({f}_{\mathrm{RF}})$ of our set-up can be found in Supplementary Fig. S1.

Simulations of ${S}^{sim}\left({V}_{\text{pk,jun}}\right)$ were performed by first convolving an idealised ${G}^{sim}({V}_{\text{DC}})$ with the probability distribution function of the arcsine distribution:

$$G^{sim} \left( {V_{{{\text{DC}}}} ,V_{{\text{pk,jun}}} } \right)|_{{\text{RF on}}} = \mathop \smallint \limits_{{ - V_{pk,jun} }}^{{ + V_{pk,jun} }} G^{sim} \left( {V_{{{\text{DC}}}} + V_{{\text{pk,jun}}} } \right)|_{{\text{RF off}}} \cdot u\left( {V_{{{\text{rf}}}} } \right) \cdot dV_{{{\text{rf}}}}$$

where $G^{sim} \left( {V_{{{\text{DC}}}} } \right)|_{{\text{RF off}}}$ is the idealised spectrum without RF, $G^{sim} \left( {V_{{{\text{DC}}}} ,V_{{\text{pk,jun}}} } \right)|_{{\text{RF on}}}$ is what the spectrum would look like with an RF modulation of amplitude of $V_{{\text{pk,jun}}}$ applied, and

$$u\left( {V_{{{\text{rf}}}} } \right) = \frac{1}{{\pi \cdot V_{{\text{pk,jun}}} \cdot \sqrt {1 - \left( {V_{{{\text{rf}}}} /V_{{\text{pk,jun}}} } \right)^{2} } }}$$

is a weight function with the shape of the probability density function of the arcsine distribution that describes the temporal average of the RF modulation of the bias voltage^22^. $G^{sim} \left( {V_{{{\text{DC}}}} } \right)|_{{\text{RF off}}}$ either takes the form of a sigmoid function centred at $V_{{{\text{DC}}}} =$ 0

$$G^{sim} \left( {V_{{{\text{DC}}}} } \right)|_{{\text{RF off}}} = \frac{a}{{1 + {\text{exp}}\left( { - V_{{{\text{DC}}}} /b} \right)}}$$

or a Gaussian distribution function centred at $V_{{{\text{DC}}}} =$ 0

$$G^{sim} \left( {V_{{{\text{DC}}}} } \right)|_{{\text{RF off}}} = a \cdot {\text{exp}}\left( {\frac{{ - V_{{{\text{DC}}}}^{2} }}{{2 \cdot \left( {b/2\sqrt {2{\text{Ln}}2} } \right)^{2} }}} \right)$$

to simulate steps and peaks, respectively. Here, $a$ is the height of the step or peak and $b$ is the width of the step or the FWHM of the peak in their respective equations. From $G^{sim} \left( {V_{{{\text{DC}}}} ,V_{{\text{pk,jun}}} } \right)|_{{\text{RF on}}}$, ${S}^{sim}\left({V}_{\text{pk,jun}}\right)$ was approximated numerically by calculating the difference between ${G}^{sim}({V}_{\text{DC}},{V}_{\text{pk,jun}}){|}_{\mathrm{RF on}}$ and ${G}^{sim}({V}_{\text{DC}},{V}_{\text{pk,jun}}+1 \mathrm{mV}){|}_{\mathrm{RF on}}$. This was done for different ${V}_{\text{pk,jun}}$, ${V}_{\text{DC}}$, as well as for different values of the parameters $a$ and $b$.

## Supplementary Information

Supplementary Information.
