# Supplementary material for: Enhanced conductance response in radio frequency scanning tunnelling microscopy
Source: Sci Rep. 2022 Apr 13;12:6183. doi: 10.1038/s41598-022-09820-7 (PMC9007990; doi:10.1038/s41598-022-09820-7)
Supplement: Supplementary file 1 — Supplementary Information. [file 41598_2022_9820_MOESM1_ESM.pdf]

Supplementary information for "Enhanced conductance response in radio frequency scanning tunnelling microscopy"

Bareld Wit[1,*], Radovan Vranik[1] & Stefan Müllegger[1]

[1] Institute of Semiconductor and Solid State Physics, Johannes Kepler University Linz, 4040, Linz, Austria. Correspondence and requests for materials should be addressed to B.W. (email: bareld.wit@jku.at)

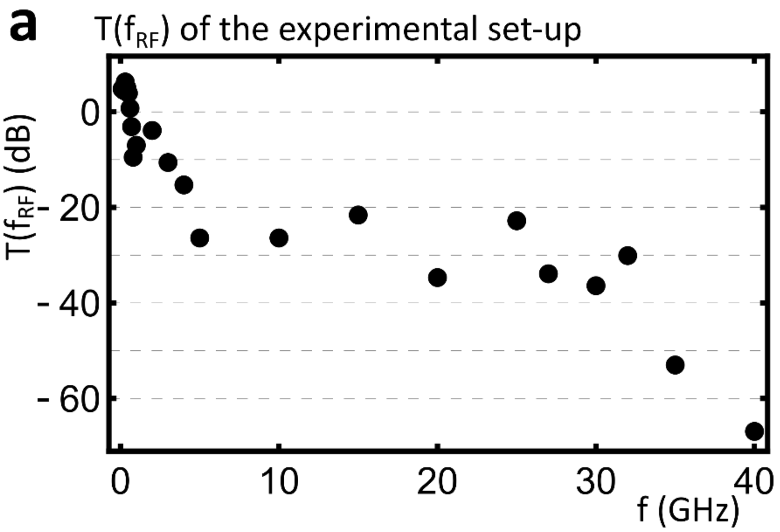

Figure S1: $T(f_{\mathrm{RF}})$ of the experimental set-up as described in the Methods section of the main text over a wide frequency range.
